# Predator-induced fear causes PTSD-like changes in the brains and behaviour of wild animals

**DOI:** 10.1038/s41598-019-47684-6

**Published:** 2019-08-07

**Authors:** Liana Y. Zanette, Emma C. Hobbs, Lauren E. Witterick, Scott A. MacDougall-Shackleton, Michael Clinchy

**Affiliations:** 1 Department of Biology, Western University, London, Ontario, N6A 5B7 Canada; 2 Department of Psychology, Western University, London, Ontario, N6A 5B7 Canada; 3 The Advanced Facility for Avian Research, Western University, London, Ontario, N6A 5B7 Canada

**Keywords:** Population dynamics, Behavioural ecology, Human behaviour, Stress and resilience

## Abstract

Predator-induced fear is both, one of the most common stressors employed in animal model studies of post-traumatic stress disorder (PTSD), and a major focus of research in ecology. There has been a growing discourse between these disciplines but no direct empirical linkage. We endeavoured to provide this empirical linkage by conducting experiments drawing upon the strengths of both disciplines. Exposure to a natural cue of predator danger (predator vocalizations), had enduring effects of at least 7 days duration involving both, a heightened sensitivity to predator danger (indicative of an enduring memory of fear), and elevated neuronal activation in both the amygdala and hippocampus – in wild birds (black-capped chickadees, *Poecile atricapillus*), exposed to natural environmental and social experiences in the 7 days following predator exposure. Our results demonstrate enduring effects on the brain and behaviour, meeting the criteria to be considered an animal model of PTSD – in a *wild* animal, which are of a nature and degree which can be anticipated could affect fecundity and survival in free-living wildlife. We suggest our findings support both the proposition that PTSD is not unnatural, and that long-lasting effects of predator-induced fear, with likely effects on fecundity and survival, are the norm in nature.

## Introduction

Biomedical scientists studying post-traumatic stress disorder (PTSD), and ecologists, have independently developed an interest in the impacts of predator-induced fear in the last two decades^1,2^. These interests are converging, with a dramatic growth in interdisciplinary discourse over the past few years, which has been detailed in multiple reviews^3–8^, and fostered at dedicated conferences^9^. Influenced by ecologists, biomedical researchers have begun to consider that PTSD may not be an unnatural, “maladaptive”, dysfunction, but rather a naturally-occurring phenomenon serving an evolutionarily adaptive purpose^3,5–7,9–12^. Ecologists in turn have begun appreciating that predator-induced fear can have long-lasting consequences transforming the animal’s subsequent reactions to predators^3,4,8,9,13,14^. This discourse having begun, the next essential step is to empirically establish that there is a linkage between the two disciplines, by demonstrating that PTSD-like changes in the brain and behaviour can occur in wild animals. Whereas there is a large literature on the behavioural effects of predator-induced fear in wild animals^2–4,8,15,16^, and a considerable number of studies have documented the effects on stress physiology (particularly glucocorticoid levels)^3,4,8^, whether predator-induced fear has enduring effects on the brain in wild animals remains to be experimentally tested^3,4,8,17^.

W. B. Canon coined the phrase “fight or flight” in 1915, to describe the immediate, transitory response of organisms to a threat^18^. A century later, we now well know that life-threatening events can have enduring effects on the brain and behaviour, not just transitory ones, as demonstrated most clearly by PTSD. Developing a medical treatment of almost any human ailment requires first developing an ‘animal model’, which typically entails inducing the condition in laboratory rodents, primarily to test the efficacy of drugs, and PTSD is no different. A recent comprehensive review of over 600 animal model studies of PTSD identified six experimental paradigms which meet the criteria of inducing neurobiological and behavioural effects, enduring from 7 to 90 days after stress termination, that mirror those seen in humans^19^. One of the most commonly-used of these paradigms not only successfully induces enduring effects, but also well-emulates the etiology of PTSD in simulating a life-threatening event; by exposing laboratory rodents to predator cues, for example, a live cat, or predator odours^1,5,6,19,20^ (as described in over 170 papers to date; Web of Science search for “predator” and “PTSD”, 21 May 2019, excluding papers referring to: humans as “predators”, or “Predator” drones).

Avoiding predation is a preeminent selective force in nature because failing to do so immediately extinguishes the individual’s future Darwinian fitness^15,16^. Retaining a powerful enduring memory of a life-threatening predator encounter is thus clearly evolutionarily beneficial if it helps the individual avoid such events in the future^3,4,8^. Contemplating this, in light of the many PTSD-like changes manifest in laboratory rodents in response to predator-induced fear^19^, has prompted a growing number of biomedical researchers to propose^3,5–7,9–11^ that “PTSD is the cost of inheriting an evolutionarily primitive mechanism that considers survival more important than the quality of one’s life”^12^. In this view, PTSD-like changes in the brain and behaviour are not unnatural or “maladaptive”, but are rather evolutionary adaptations which entail costs, such as “hypervigilance”^12,19,20^ and the avoidance of trauma-related cues^19^, that provide the benefit of increasing the probability of survival, by increasing the likelihood of detecting a life-threatening danger (hypervigilance), and reducing the probability of encountering one (avoidance). In humans, the costs in terms of reduced quality of life resulting from hypervigilance and avoidance of trauma-related cues, can be numerous and diverse^3,5–7,9–12^. In wild animals, one of the most well-established principles in ecology is that the cost of increased vigilance is reduced time spent feeding, and avoiding predators generally also entails a significant cost with respect to reducing feeding opportunities^2,15,16,21^.

The cost of evolutionarily prioritizing avoiding predation underlies both this recent thinking about PTSD, and recent thinking about predator-prey ecology. What is termed the “ecology of fear”^2^ concerns quantifying the total impact of predators on prey populations and communities. The traditional view in ecology is that predators directly kill prey, thereby reducing prey survival, and this is the limit of their impact. The ‘ecology of fear’ posits that the behavioural, physiological and neurobiological costs of avoiding predation (‘fear’ for short^2,18,22^), such as reduced feeding time or reduced feeding opportunities, may additionally reduce prey fecundity and survival, and the total reduction in prey numbers resulting from exposure to predators may thus far exceed that due to direct killing alone^4,21^. Two factors have limited the general acceptance of this. The first is that prey responses to predators in the wild are still predominantly assumed to be instantaneous and fleeting (i.e., “fight or flight”), and thus not sufficiently long-lasting to affect fecundity and survival^3,4,8^. Fecundity, for example, is unlikely to be affected by an animal missing a meal because it fled from a predator; only an enduring, protracted period of reduced feeding is likely to reduce fecundity, and it is thus necessary to demonstrate that predator-induced fear can have enduring effects^3,4,8^. The second factor that has limited the general acceptance of the idea that fear can affect fecundity and survival results from the fact that, whereas one can watch a predator killing a prey one cannot ‘see’ fear killing a prey, but must instead infer its effects; meaning that manipulative experiments are essential to making strong inferences about the effects of fear. Due to the logistical challenges involved only a handful of recent experiments have demonstrated that predator-induced fear can reduce prey fecundity and survival in free-living wildlife, but these have nonetheless established that the effects of fear itself (the costs of avoiding predation) can be powerful enough to reduce the number of young born and surviving to adulthood by more than 50%^23–29^.

To experimentally test if predator-induced fear causes PTSD-like changes in the brains and behaviour of wild animals we drew upon the strengths of the two disciplines involved. From animal model studies of PTSD, we adhered to the criteria of testing for effects enduring for at least 7 days, affecting behaviour (“hypervigilance”^12,20^), and the brain areas (amygdala and hippocampus), most pertinent to PTSD in humans^1,12,19,20^. We experimentally tested for these enduring effects by employing a well-established predator-fear protocol used in animal model studies of PTSD (2 days experimental predator cue exposure followed by 7 days without^30,31^), and subsequently measuring both, a behavioural reaction to danger (‘freezing’, i.e., time spent ‘vigilant and immobile’^15,16^) commonly assessed in animal model studies of PTSD^19^, and a well-studied marker of long-term neuronal activation (∆FosB^30–32^). To maximize the ecological relevance we tested for effects on birds (black-capped chickadees, *Poecile atricapillus*), because we knew that an enduring effect of 7 days duration on the behaviour of birds can affect survival, from the field experiments conducted to date demonstrating that predator-induced fear can reduce fecundity and survival in free-living wildlife^25,29^. We further enhanced the external validity by inducing fear using predator vocalizations (as done in most of the aforesaid field experiments), housing the birds outdoors in flocks for the 7 days after the 2 days of experimental cue exposure (to determine if effects were measurable after a week of natural experiences), and assessing their enduring memory of fear by evaluating their reaction to another, different, natural cue of predator danger (conspecific alarm calls^33,34^). Our results demonstrate that PTSD-like changes in the brain and behaviour can occur in wild animals; which we suggest supports both the proposition that PTSD is not unnatural^3,5–7,9–12^, and that long-lasting effects of predator-induced fear, with likely effects on fecundity and survival, are the norm in nature^2–4,8,21,25,29^.

## Results

### Enduring effect on behaviour

**Figure 1:**
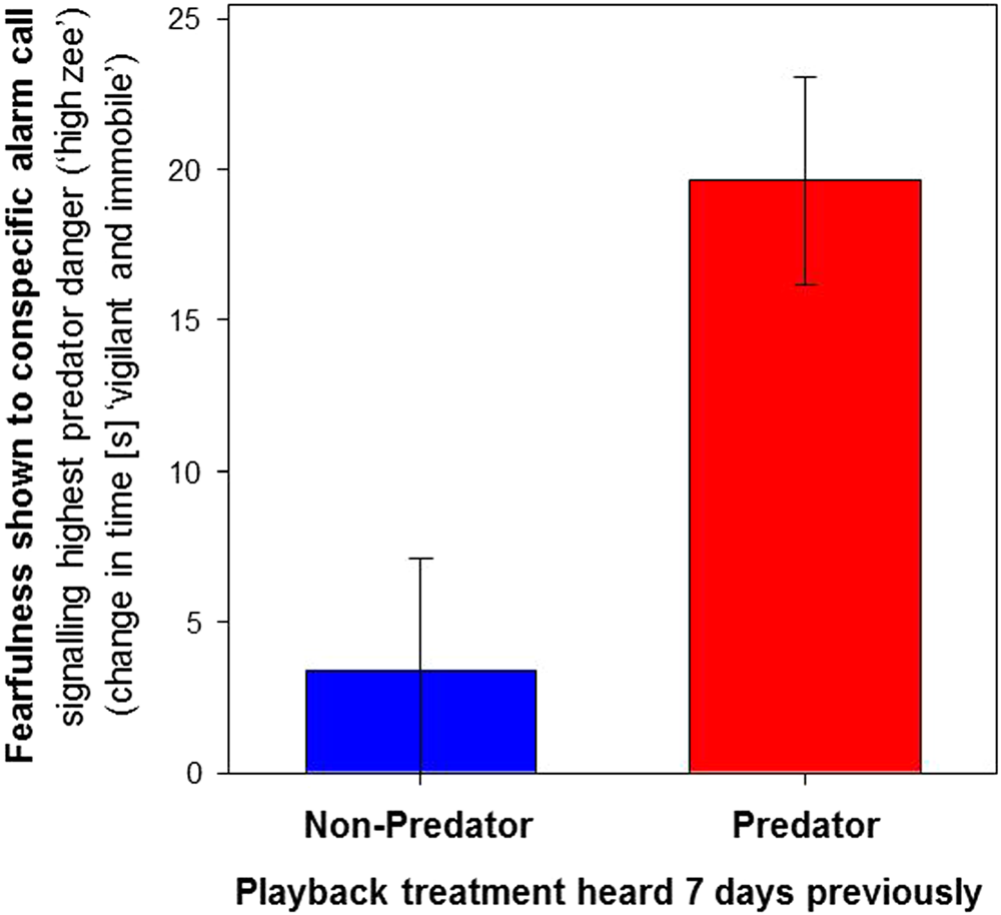
Effect of predator (red) and non-predator (blue) playbacks heard 7 days previously on the fearfulness shown in response to hearing conspecific alarm calls signalling the highest level of predator danger (‘high zee’ calls), as quantified by the change in time (seconds) spent ‘vigilant and immobile’, compared between the 1 minute before, vs. the 1 minute after, the start of the first alarm call. Values are means ± S.E.

Exposure to predator cues had an enduring effect on behaviour of at least 7 days duration consistent with having induced an enduring memory of fear. In response to hearing conspecific alarm calls signalling the highest level of predator danger (‘high zee’ calls)^33,34^, individuals that heard predator vocalizations 7 days previously behaved significantly more fearfully, demonstrating a 6-fold greater increase in time spent ‘vigilant and immobile’ (i.e., ‘freezing’^15,16,19,33^) than did individuals that heard non-predator vocalizations 7 days previously (Fig. 1; *F*_1,11_ = 10.8, P = 0.007, n = 8 predator and 7 non-predator individuals).

### Enduring effects on neuronal activation

**Figure 2:**
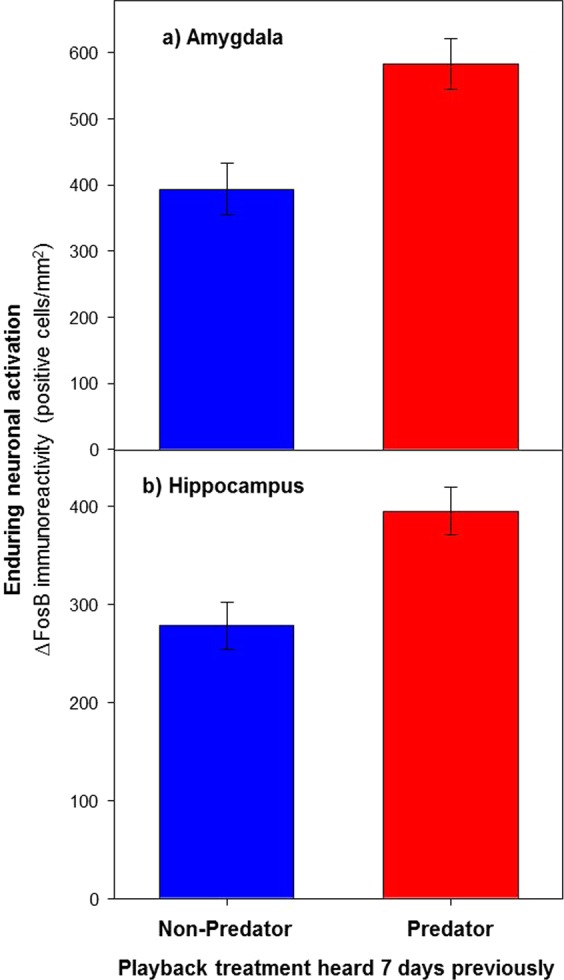
Effect of predator (red) and non-predator (blue) playbacks heard 7 days previously on enduring neuronal activation in (**a**) the amygdala and (**b**) hippocampus, as quantified by ∆FosB immunoreactivity (positive cells/mm^2^). Values are means ± S.E.

Predator-induced fear had enduring effects on neuronal activation of at least 7 days duration in both the amygdala (nucleus taeniae of the amygdala, see Methods) and hippocampus. Individuals that heard predator vocalizations 7 days previously demonstrated a highly significant (Fig. 2a; *F*_1,8_ = 21.0, P = 0.002), 48% greater level of ΔFosB immunoreactivity (positive cells/mm^2^) in the amygdala than did those that heard non-predator vocalizations 7 days previously, as well as a highly significant (Fig. 2b; *F*_1,8_ = 12.1, P = 0.008), 42% greater level of ΔFosB immunoreactivity in the hippocampus (n = 6 predator and 6 non-predator individuals).

### Immediate effects on neuronal activation

**Figure 3:**
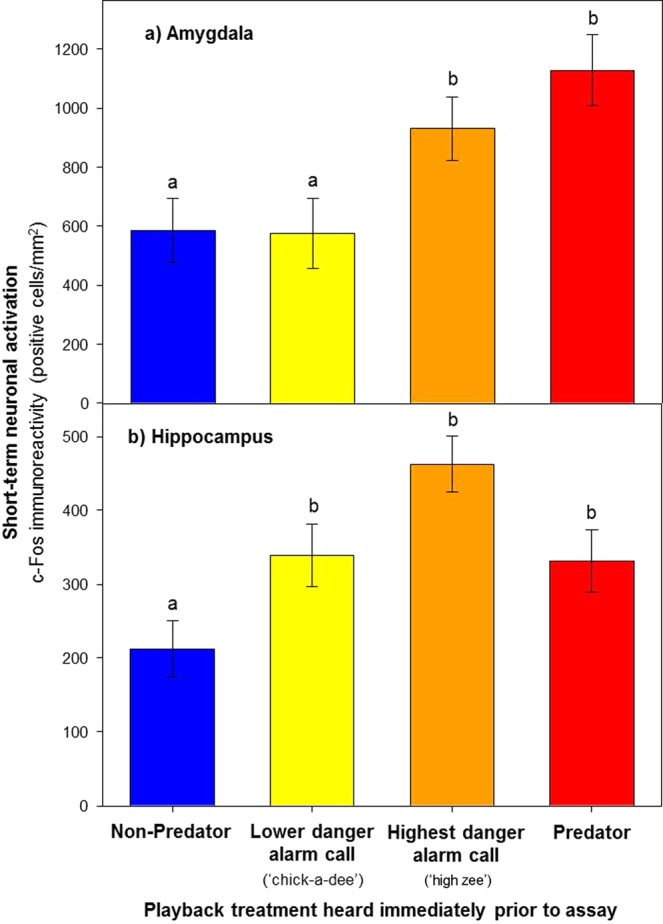
Effect of immediately previously heard playbacks of, predator (red) and non-predator (blue) vocalizations, and conspecific alarm calls signalling the highest level of predator danger (‘high zee’ calls; orange) and a lower level of predator danger (‘chick-a-dee’ calls; yellow), on short-term neuronal activation in (**a**) the amygdala and (**b**) hippocampus, as quantified by c-Fos immunoreactivity (positive cells/mm^2^). Letters indicate significant differences (*p* < 0.05) in Dunnett’s tests comparing the other treatments vs. the control (non-predator). Values are means ± S.E.

The effects on the brain and behaviour found in the main experiment were directly attributable to the fear induced by hearing the various audio playbacks, as demonstrated by the results from a subsidiary experiment testing the immediate effects of hearing the various playbacks on short-term neuronal activation (90 minutes after experimental cue exposure). In our subsidiary experiment, there was a highly significant overall effect of playback treatment on the level of c-Fos immunoreactivity in both the amygdala (Fig. 3a; *F*_3,14_ = 5.7, P = 0.009) and hippocampus (Fig. 3b; *F*_3,14_ = 8.4, P = 0.002). Hearing predator vocalizations (n = 5 individuals) significantly increased the level of c-Fos immunoreactivity in both the amygdala (Fig. 3a; Dunnett’s test, P = 0.013) and hippocampus (Fig. 3b; Dunnett’s test, P = 0.027), in comparison to hearing non-predator vocalizations (n = 5 individuals). Consistent with ‘high zee’ conspecific alarm calls signalling the highest level of predator danger^33,34^, individuals that heard this type of alarm call (n = 5 individuals) similarly demonstrated significantly increased immunoreactivity in both the amygdala (Fig. 3a; Dunnett’s test, P = 0.046) and hippocampus (Fig. 3b; Dunnett’s test, P = 0.001). In contrast, individuals (n = 5) that heard alarm calls signalling a lower level of predator danger (‘chick-a-dee’ calls)^33,34^, while demonstrating significantly increased c-Fos immunoreactivity in the hippocampus (Fig. 3b; Dunnett’s test, P = 0.041), showed no corresponding effect whatsoever in the amygdala (Fig. 3a; Dunnett’s test, P = 0.864).

## Discussion

Our demonstration of effects of predator-induced fear on the brain and behaviour enduring at least 7 days, meets the criteria to be considered an animal model of PTSD^19^ – in a *wild* animal. Individuals exposed to predator cues manifested an enduring memory of fear, demonstrating a heightened sensitivity to predator danger rather than a memory of specific cues, as shown by their greater responsiveness to a cue of predator danger distinct from those they were exposed to 7 days previously. “Hypervigilance” is one of the characteristic consequences of PTSD in humans^12,19,20^, and inducing an enduring exaggerated fear response is accordingly one of the criteria it is necessary to meet to be considered an animal model of PTSD, which our results satisfy^19^. Enduring neurobiological effects, on the “fear-circuit” involving the amygdala^19^ and the hippocampus, are also criteria which our results meet^1,12,19,20^. That these enduring effects on the amygdala and hippocampus were directly attributable to the fear induced by hearing predator cues 7 days previously, is clear from the immediate activation of these brain areas resulting from hearing these cues, as shown in our subsidiary experiment. Demonstrating that predator-induced fear can cause PTSD-like changes in the brain and behaviour in wild animals establishes the empirical linkage between their disciplines, which growing numbers of biomedical scientists studying PTSD, and ecologists, are recognizing exists^3–9^.

To our knowledge ours is the first experiment to demonstrate that predator-induced fear can cause enduring effects on the amygdala and hippocampus in a wild animal. We suggest this is solely due to the newness of this field of research, and more studies will reveal such effects to be the norm in nature. Indeed, the striking correspondence between the effects we have demonstrated in a wild bird responding to acoustic predator cues, and those found in mammals (laboratory rodents) reacting to visual and olfactory cues in animal model studies of PTSD^19^, testifies to the likely generality. This correspondence extends further, in relation to the cue inducing enduring effects being a life-threatening one, consistent with the etiology of PTSD. Our subsidiary experiment demonstrated that hearing predator vocalizations, and high threat (‘high zee’) alarm calls, both had significant effects on immediate neuronal activation in both the amygdala and hippocampus (Fig. 3), whereas hearing low threat (‘chick-a-dee’) alarm calls did not affect immediate activation in the amygdala (Fig. 3a) and hence is unlikely to induce enduring effects. The ‘chick-a-dee’ alarm call is emitted when mobbing a predator, which entails a group of prey attacking a predator in a position (e.g. perched) such that it poses little danger^33,34^. Our results correspond with recent studies reporting immediate activation in the amygdala in birds shown a life-threatening cue, but not a lower threat one (a perched hawk)^35,36^. We suggest the fact that the enduring effects on the brain we have shown in a wild animal appear restricted to life-threatening predator cues, supports the proposition that the enduring effects in PTSD are the cost of evolutionarily prioritizing survival^12^.

The effectiveness of the ‘predator exposure’ paradigm in animal model studies of PTSD has been proposed to be attributable to the presentation of a cue of predator danger, which is “inescapable”^19^. In the context of the ‘predator exposure’ paradigm, we suggest ‘inescapability’ *per se* may not be pertinent to predator exposure’s causing PTSD-like changes, but rather what is, is the intensity of the fear induced. In our experiments predator exposure was ‘inescapable’ because the subjects were in small cages during the cue exposure period. Predator cue exposure and ‘inescapability’ in combination did not necessarily cause effects, as shown by the lack of amygdala activation in response to the low threat cue (‘chick-a-dee’ alarm; Fig. 3a). This corresponds with the recent finding of a lack of amygdala activation to a low threat visual cue (perched hawk), which too was ‘inescapable’, because the subjects were caged^36^.

Paradigms other than predator exposure are used in animal model studies of PTSD and these include “immobilization or restraint stress” and “inescapable shocks”^19^. Thus, immobility and inescapability are traumatic for laboratory rodents. For wild animals, fleeing is generally the principal response to a predator threat^16^, and the relative immobility and inescapability resulting from being in captivity could be largely responsible for the strength of the effects documented in experiments testing the consequences of predator exposure on wild animals in captivity^3,4,8^. Consequently, establishing that the fear of predators, separate from the stress of captivity, or capture, can have enduring effects on the brains and behaviour of wild animals comparable to those in laboratory rodent studies of PTSD – and those demonstrated here – requires determining if such effects are demonstrated in free-living wildlife in nature, something which remains to be tested experimentally^3,4,8^. Our results provide a necessary first step towards the goal of testing the enduring effects of fear in free-living wildlife. Free-living wild animals regularly experience intensely life-threatening predator encounters and are frequently physically traumatized as a result. Most predator attacks are unsuccessful^37,38^, meaning most prey escape, but they do not necessarily escape unharmed. For example, recent studies have shown that up to 32% of living adult female giraffes (*Giraffa camelopardalis*) bear scars from lions (*Panthera leo*)^39^, 25% of harbour porpoises (*Phocoena phocoena*) bear claw and bite marks from grey seals (*Halichoerus grypus*)^40^, and 100% of manta rays (*Manta alfredi*) bear multiple bite wounds from sharks^41^. From an evolutionary perspective, it is difficult to envisage that these free-living wild animals do not bear enduring psychological effects, corresponding with their physical injuries^3,4,8,9,16^.

Whether the enduring effects in PTSD are ‘natural’ or not has a bearing on both biomedical research and clinical practice^3,5–7,9–12^. Our results demonstrating enduring amygdala ‘hyperactivation’^12,19^ – in a wild animal, in response to natural predator cues, which persists after a period of natural experiences, and is associated with heightened sensitivity to subsequent natural predator cues, is all directly relevant to recent discourse among biomedical researchers regarding whether the amygdala in PTSD is “hyperfunctional” or dysfunctional^12,19^. The ‘hyperfunctional’ view is that the amygdala is functioning perfectly naturally in being ‘switched on’ by a life-threatening event, in anticipation of a subsequent one^12,42^, whereas the ‘dysfunctional’ view is that the amygdala is damaged or diseased^19,42^; reducing amygdala hyperactivity being the appropriate treatment objective in the former view, in contrast to the latter’s focus on ‘blocking’ activation (pharmacologically or otherwise)^43^. Clinically, psychotherapy remains the most effective treatment for PTSD, rather than pharmacotherapy^44^. Particularly among military veterans, PTSD is associated with a sense of shame, which can often lead to suicide^45^. Recent psychotherapeutic approaches (e.g. “compassion focused therapy”^46^) aim to alleviate the sufferer’s shame by helping them understand their symptoms within the context of the evident evolutionary functions of those symptoms, such as the survival benefits of hypervigilance in anticipation of a subsequent life-threatening event^12,42,46,47^. Evidence indicating that PTSD is not ‘unnatural’ but rather a cost of evolutionarily prioritizing survival^3,5–7,9–12,42,43,46,47^, thus directly supports such therapeutic approaches.

Our results demonstrate that predator-induced fear in wild animals can entail more than just “fight or flight”, and instead can produce long-lasting effects likely to affect fecundity and survival. We tested for enduring effects of predator-induced fear on the brains and behaviour of wild birds, because birds have been the subjects of all the experiments to date demonstrating that predator-induced fear can affect fecundity and survival in free-living wildlife^23–29^. There is abundant evidence to indicate that comparable effects occur in mammals, and most other animals^2,4,8,9,15,16,21^, and the present lack of field experiments on mammals is most likely due to the logistical difficulties resulting from mammals typically being secretive and nocturnal. Our experiment induced a heightened sensitivity to predator cues, which endured at least 7 days, and involved increased time spent vigilant (Fig. 1). Heightened sensitivity to predator cues lasting at least 7 days, like protracted exposure to them^25^, has been shown to impair parental care and reduce offspring survival in free-living wild birds. This was established in a recent study which assessed individual differences in fearfulness (heightened sensitivity) among parental birds, in their reaction to short-term (1 h) playbacks of predator cues (much as we did in our behavioural assay), and then showed that this measure of each parent’s fearfulness predicted their offspring’s survival to independence, 3 weeks later^29^. Importantly, it was the responses of more fearful parents to entirely naturally-occurring predator cues, over this 3 weeks, which evidently led to the deaths of their offspring, indicating that enduring heightened sensitivity to predator danger such as we have shown, can be expected to affect fecundity and survival in nature.

For wild animals the cost of avoiding predation can include parents having some of their offspring die, because the time taken being vigilant and immobile in response to predator cues prevents the parent from having the time to find enough food to feed all of its young^25,29^. Critically, it must be remembered that the cost of *failing* to avoid predation, i.e., the parent’s death in this example, would likely entail all of its offspring dying not just some, and the parent obviously never having any more^2,15,16^. Evolutionarily prioritizing survival at the cost of the ‘quality of life’ is thus part of nature, and the grim arithmetic in the face of predation risk exemplified by parents ensuring their survival and that of some of their offspring at the cost of others, almost certainly applied equally to humans, as to most animals, throughout our prehistory^12^. Prioritizing survival in the face of predation risk has only very recently, in evolutionary terms, become less immediately relevant and universal to humans, thanks to our progressively having destroyed almost all of our predators (large carnivores), in just the past few centuries and decades^48,49^. Considered ecologically, a species (ourselves) now living largely free of predation risk is what is highly anomalous^50^, rather than the arguably evolutionarily adaptive response seen in PTSD. Having established the empirical linkage between animal model studies of PTSD and ecology, we view our results not as a final word, but a starting point, in the advance from the current interdisciplinary discourse between biomedical scientists studying PTSD and ecologists, to a new fully-fledged interdisciplinary field of research exploring the relevance of predator-induced fear in relation to both ourselves and other animals.

## Materials and Methods

### Overview of experimental design

Wild free-living adult chickadees of both sexes were live-captured during the non-breeding season and housed in flocks outdoors for 1 week prior to experimental trials. To experimentally test if predator-induced fear has enduring effects, individuals were housed solitarily in acoustic isolation chambers and exposed for 2 days to audio playbacks of the vocalizations of either predators (treatment group) or non-predators (control group)^23,25–27,29–31^, and then housed again in flocks outdoors for 7 days, during which time they were not exposed to any further experimental cues, but were instead exposed to natural sights and sounds and social interactions. Enduring effects on behaviour were then assessed in one set of individuals and effects on the brain were evaluated in a separate set, to ensure that effects on the brain were attributable to exposure to the cues heard 7 days previously and not the cues used in assessing behaviour.

To assess effects on behaviour, individuals were again housed solitarily in acoustic isolation chambers, and all were exposed for 15 minutes to playbacks of conspecific alarm calls (‘high zee’ calls^33,34^), a signal which, like hearing predator vocalizations, alerts the hearer to a predator danger^33,34^, but in the context of the experiment entailed individuals hearing cues (chickadee vocalizations) distinct from those they were exposed to 7 days previously. More fearful reactions in those individuals that previously heard predators, vs. non-predators, could thereby be interpreted as reflecting an enduring memory of fear, i.e., a heightened sensitivity to predator danger^19^, rather than a memory of the specific cues heard 7 days previously. To gauge the fearfulness of their reactions we measured the time each individual remained ‘vigilant and immobile’ (i.e., ‘freezing’) upon first hearing the alarm calls; ‘freezing’ being an anti-predator behaviour demonstrated in almost every type of animal^15,16,33^, which is commonly measured in animal model studies of PTSD^19^.

To determine if there were enduring effects on the brain we assayed ∆FosB expression to identify long-term neuronal activation^30–32^ in the avian homologues of the two brain regions most pertinent to PTSD in humans, the amygdala and hippocampus^12,19,20,51,52^. The amygdala is responsible for fear processing and the acquisition and expression of fear memories, as demonstrated by lesioning studies on laboratory rodents, and the fact that people with a damaged amygdala report not feeling fearful in response to a variety of fear-provoking stimuli, including life-threatening traumatic events^51–54^. The hippocampus is involved in forming declarative, episodic and spatial memories^51,52^. Whereas amygdala activation generally increases with the intensity of a trauma, the duration and magnitude of effects on the hippocampus can be complex and vary with what is measured^51,52^. ∆FosB is a protein produced by the *FosB* gene. It is a transcription factor, meaning it modifies the transcription of other genes. Whereas most transcription factors degrade within hours, ∆FosB is unusually stable and can continue to have effects for weeks, effects which include promoting resistance to the deleterious consequences of chronic stress^32^.

To be certain that enduring effects on the brain and behaviour, found in the main experiment, were directly attributable to the fear induced by hearing the various audio playbacks, we conducted a subsidiary experiment, testing the immediate effects of hearing predator vocalizations and conspecific alarm calls on short-term neuronal activation in the same brain areas examined in the main experiment. Additional wild free-living adult chickadees of both sexes were live-captured during the non-breeding season, housed in flocks outdoors for 1 week, and then housed solitarily in acoustic isolation chambers and exposed for 30 minutes to audio playbacks of either predator vocalizations, conspecific alarm calls signalling the highest level of predator danger (‘high zee’ calls^33,34^) or a lower level of predator danger (‘chick-a-dee’ calls^33,34^), or non-predator vocalizations. To quantify the immediate effects on short-term neuronal activation we assayed c-Fos expression in the two relevant brain areas; c-Fos being a short-lived transcription factor that degrades in a few hours, which is related to ∆FosB^32^.

### Species, housing and playback procedures

The black-capped chickadee is a small (12 g) songbird resident year-round throughout southern Canada, which lives in small territorial flocks over winter. Male and female chickadees look and sound alike, and in the non-breeding season their behaviour is indistinguishable, such that the sexes can only be discriminated by a slight difference in wing length^33^. We included both sexes in our experiment for the purposes of obtaining an ecologically representative sample rather than to compare between the sexes, which we expected would not likely differ in their reactions to predators^33^. The immediate anti-predator responses of chickadees to playbacks of predator vocalizations and conspecific alarm calls have been well-studied^33,34^, as have the short-term neurobiological effects on the auditory processing areas of the brain resulting from hearing these signals of predator danger^55^.

Wild free-living chickadees were live-captured on the campus of Western University (London, Ontario, Canada) in the non-breeding season (September to March), and housed at the University’s Advanced Facility for Avian Research (http://birds.uwo.ca). Upon initial capture, and during the 7 days following the 2 days of cue exposure in the main experiment, individuals were housed in flocks in room-sized (2.1 × 2.4 × 3.7 m) outdoor aviaries on the Facility’s roof. During cue exposure individuals were housed solitarily, in small cages (25 × 30 × 37 cm), inside acoustic isolation chambers, to ensure that all they heard were the experimental cues.

In the main experiment, 27 individuals (15 males, 12 females) heard, during the 2 days of cue exposure, playlists matched for maximum amplitude and frequency and average decibel level, composed of the vocalizations of either, six predator species known to prey on chickadees (Cooper’s hawk, *Accipiter cooperii*; sharp-shinned hawk, *Accipiter striatus*; northern saw-whet owl, *Aegolius acadicus*; red-tailed hawk, *Buteo jamaicensis*; merlin, *Falco columbarius*; barred owl, *Strix varia*), or six non-threatening non-predator species (mallard, *Anas platyrhynchos*; wood frog, *Lithobates sylvaticus*; song sparrow, *Melospiza melodia*; downy woodpecker, *Picoides pubescens*; hairy woodpecker, *Picoides villosus*; red-breasted nuthatch, *Sitta canadensis*). All of these predator and non-predator species occur locally and their vocalizations would all be heard naturally by chickadees in the area. Vocalizations were broadcast for a total of 5 minutes per hour, during the 12 hours of daylight, at randomly selected intervals, with each species used one to four times every two hours, using different exemplars and call lengths each time. Individuals were randomly-assigned to treatment, balancing assignment between the treatments and sexes. To assess the enduring effects on behaviour, 7 days after predator cue exposure, 15 individuals were randomly-selected, balancing between the treatments and sexes, and exposed to playbacks of conspecific alarm calls signalling the highest level of predator danger (‘high zee’ calls^33,34^), for 15 seconds every minute, over a period of 15 minutes. All wild chickadees are familiar with conspecific alarm calls^33,34^. Individuals were filmed for 15 minutes before and during cue exposure, and the time they spent ‘freezing’^15,16,19,33^, operationally defined as immobile (stationary; not hopping, walking or flying) with their head upright and eyes open (vigilant; as opposed to, e.g. head down foraging)^33^, in the 1 minute before and after the start of the first playback^33,56,57^, was subsequently scored by an observer blind to which treatment each bird had received 7 days previously.

In the subsidiary experiment testing the immediate effects of hearing the playbacks on short-term neuronal activation, 22 individuals were randomly-assigned to treatment, balancing between treatments and sexes, and heard playbacks broadcast for 15 seconds every minute, over a period of 30 minutes, consisting of either predator (northern saw-whet owl) vocalizations, conspecific alarm calls (‘high zee’ or ‘chick-a-dee’ calls^33,34^) or non-predator (red-breasted nuthatch) vocalizations. Individuals hearing conspecific alarm calls necessarily heard the vocalizations of a single species (chickadees), which we matched by broadcasting a single predator and non-predator to the other individuals.

Predator and non-predator vocalizations were obtained from the Macaulay Library (www.macaulaylibrary.org) or xeno-canto (www.xeno-canto.org). Chickadee ‘high zee’ and ‘chick-a-dee’ alarm calls were obtained by recording captive wild-caught chickadees reacting to a taxidermic mount of a northern saw-whet owl. At least three exemplars of every vocalization were utilized. All sounds were edited using Audacity (www.audacityteam.org) to eliminate noise and standardize decibel levels. All playbacks were broadcast at a volume of 74 dB using all the same make and model of speaker and mp3 player.

This research was approved by Western University’s Animal Care Committee under protocol 2010-0245 and by Environment Canada under Scientific Permit CA-0244. All procedures were conducted in licensed and inspected facilities, and followed the guidelines set forth by the Canadian Council on Animal Care.

### Neurobiological details and procedures

The nucleus taeniae of the amygdala (TnA) is the avian homologue of the mammalian medial amygdala^35,36,58^, and the hippocampus is homologous in birds and mammals^35,36,59^. To avoid any confusion concerning the relevance of our results to existing (i.e., mammalian) animal model studies of PTSD, we refer simply to the ‘amygdala’ when describing effects found in the nucleus taeniae of the amygdala. Immediate increases in activation in both the amygdala and the hippocampus have been reported previously in birds shown life-threatening visual cues^35,36^.

To assay ∆FosB and c-Fos expression in the amygdala and hippocampus in response to the audio playbacks used in our experiments, individuals were euthanized 7 days (∆FosB) or 90 minutes (c-Fos) after experimental cue exposure, using isoflurane, and perfused with 0.1 M phosphate buffered saline (pH 7.4) followed by 4% paraformaldehyde. Brains were removed, placed in 4% paraformaldehyde for a minimum of 24 h, then 30% sucrose for 24 h until saturated, and frozen at −80 °C. Brains were sectioned into 40 μm coronal slices using a cryostat at −20 °C. A series of sections were collected for Nissl staining to locate the brain regions of interest (see Supplementary Fig. S1), and two series were collected for immunohistochemistry. ∆FosB and c-Fos were labelled using commercial antibodies (Santa Cruz Biotechnology: FosB (102) rabbit IgG, sc-48; c-Fos (4) rabbit IgG, sc-52e) following established protocols^30,31,60^. Sections were processed free-floating in tissue culture wells. Sections were blocked in 0.05% H_2_O_2_ followed by 10% normal serum and then incubated in the primary antibodies diluted (1:500) in phosphate buffered saline and 0.3% Triton (PBS/T). Following incubation in the primary antibody, sections were incubated in a biotinylated secondary antibody (diluted 1:500) followed by an avidin-biotin reaction (Vectastain Elite kit PK-6100, Vector Labs). Finally, immunoreactive cells were visualized using 3,3′-diaminobenzidine tetrachloride (SigmaFAST DAB). Sections were then mounted on microscope slides, dehydrated, cleared, and cover-slipped.

We captured z-stack images of each region of interest using a Leica Digital CCD (model 420D) camera mounted on a Leica DM5000B light microscope through X10 (amygdala) and X5 (hippocampus) objective lenses. We used Leica Application Suite to compile each picture as a z-stack from a series of images taken at a regular interval (0.63 mm) throughout the focal depth of the section to create images in which all cells were in focus^60^. The area of each region was measured in mm^2^ using ImageJ software calibrated to the relevant magnification. Each image was next converted from colour to 16-bit grayscale, the background subtracted and contrast enhanced, following which the ImageJ thresholding tool was used to convert ΔFosB or c-Fos positive nuclei to black against a white background, and the ImageJ count function was employed to quantify the number of positive cells/mm^2^ in each slice in each brain region of interest (see Supplementary Fig. S2). All image processing was conducted by an observer blind to which treatment each individual had received.

We confirmed that the enduring effects on ∆FosB expression in the amygdala and hippocampus demonstrated in response to predator-induced fear (Fig. 2) were specific to these regions, by additionally assaying ∆FosB expression in the medial caudal nidopallium (see Supplementary Fig. S1), an auditory processing area in the songbird brain^55^. We followed all the same procedures as just described regarding assaying ∆FosB expression in the amygdala and hippocampus 7 days after experimental cue exposure. In contrast to the significant effects seen in the amygdala and hippocampus (Fig. 2), there was no evidence of an enduring treatment effect on ∆FosB expression in the medial caudal nidopallium (*F*_1,8_ = 1.3, P = 0.279, n = 6 predator and 6 non-predator individuals).

### Statistical analyses

We conducted two-way ANOVAs with playback treatment and sex as fixed factors. In our test of enduring effects on behaviour our dependent variable was the change in time each individual spent ‘vigilant and immobile’, in the 1 minute before, vs. the 1 minute after, the start of the first conspecific alarm call; this being a repeated-measures value, which thereby controls for individual differences in fearfulness^29,56,57^. In our tests of effects on neuronal activation we calculated averages per individual of the number of ΔFosB or c-Fos immunoreactive cells/mm^2^ across all slices, in each brain area, and used these averages in our analyses. In testing effects on neuronal activation we conducted separate ANOVAs on each brain region (amygdala and hippocampus). Following our ANOVAs concerning the short-term effects on neuronal activation of hearing the various playback treatments in the subsidiary experiment, we conducted Dunnett’s post-hoc tests comparing each treatment with the control (non-predator vocalizations). Prior to analysis, all data were Box–Cox transformed and tested for normality and homogeneity of variances. All descriptive results reported (means ± SE) are untransformed or back transformed to the original units. Analogous to statistically controlling for individual variation by analyzing the change in each individual’s response in our test for an enduring treatment effect on behaviour, we included sex in our analyses to statistically account for this as a potential source of individual variation. There were no significant sex or treatment by sex effects (all *p* > 0.30), and we accordingly only report treatment effects in the *Results*.

## Supplementary information

Supplementary Information Predator-induced fear causes PTSD-like changes in the brains and behaviour of wild animals

## Data Availability

Relevant data is provided as Supplementary Information.
