# Supplementary material for: Predator-induced fear causes PTSD-like changes in the brains and behaviour of wild animals
Source: Sci Rep. 2019 Aug 7;9:11474. doi: 10.1038/s41598-019-47684-6 (PMC6685979; doi:10.1038/s41598-019-47684-6)
Supplement: Supplementary file 1 — Supplementary Information Predator-induced fear causes PTSD-like changes in the brains and behaviour of wild animals [file 41598_2019_47684_MOESM1_ESM.docx]

**Supplementary Information**

**Predator-induced fear causes PTSD-like changes in the brains and behaviour of wild animals**

Liana Y. Zanette, Emma C. Hobbs, Lauren E. Witterick, Scott A. MacDougall-Shackleton & Michael Clinchy

**Supplementary Online Figures**

**
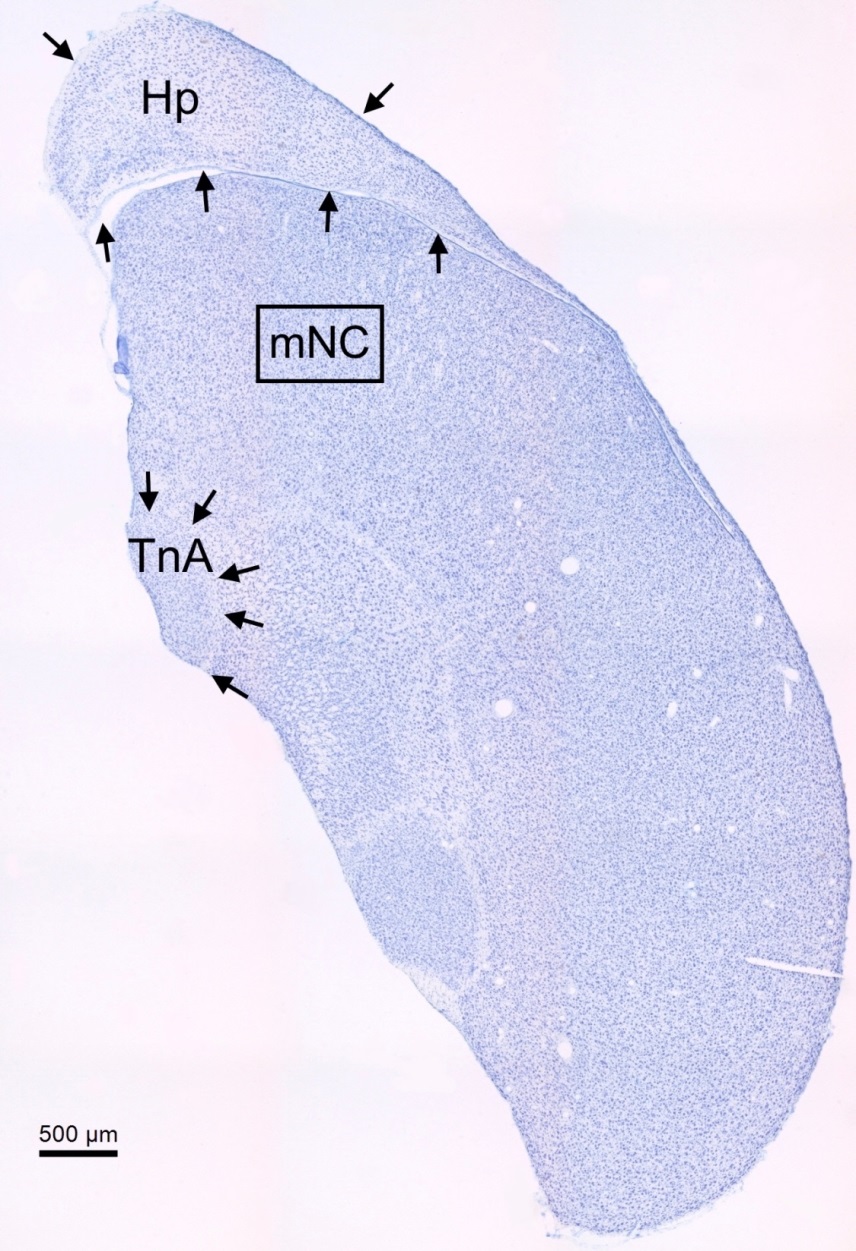
**

**Figure S1.** Nissl stained coronal section of a black-capped chickadee (*Poecile atricapillus*) brain identifying the locations of the amygdala (nucleus taeniae of the amygdala, TnA), hippocampus (Hp) and medial caudal nidopallium (mNC).

**Supplementary Information**

**Predator-induced fear causes PTSD-like changes in the brains and behaviour of wild animals**

Liana Y. Zanette, Emma C. Hobbs, Lauren E. Witterick, Scott A. MacDougall-Shackleton & Michael Clinchy

**Supplementary Online Figures**

**
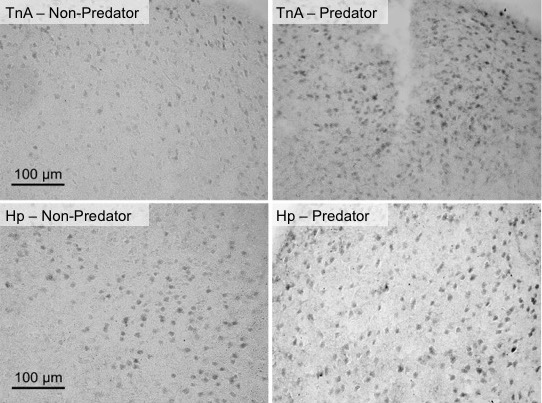
**

**Figure S2.** Examples of ΔFosB immunoreactivity in the amygdala (TnA) and hippocampus (Hp) compared between subjects that heard non-predator or predator playbacks 7 days previously.

**Supplementary Information**

**Predator-induced fear causes PTSD-like changes in the brains and behaviour of wild animals**

Liana Y. Zanette, Emma C. Hobbs, Lauren E. Witterick, Scott A. MacDougall-Shackleton & Michael Clinchy

| **Behavioural data** | | | | |
| --- | --- | --- | --- | --- |
|  |  | **Time spent vigilant and immobile (seconds)** | | |
| **Treatment** | **Sex** | **1 min before playback** | **1 min after playback** | **Change in time** |
| Non-predator | M | 29.1 | 31.1 | 2.0 |
| Non-predator | F | 17.1 | 38.7 | 21.6 |
| Non-predator | F | 36.4 | 37.2 | 0.8 |
| Non-predator | M | 27.2 | 30.9 | 3.7 |
| Non-predator | F | 60.0 | 60.0 | 0.0 |
| Non-predator | M | 32.8 | 28.6 | -4.2 |
| Non-predator | M | 60.0 | 60.0 | 0.0 |
| Predator | M | 32.5 | 60.0 | 27.5 |
| Predator | F | 36.9 | 59.8 | 22.9 |
| Predator | M | 25.2 | 59.9 | 34.7 |
| Predator | M | 39.4 | 59.8 | 20.4 |
| Predator | F | 31.1 | 59.9 | 28.8 |
| Predator | M | 34.6 | 41.5 | 6.9 |
| Predator | F | 27.1 | 36.5 | 9.4 |
| Predator | M | 34.4 | 40.8 | 6.4 |

| **Enduring neuronal activation data (ΔFosB immunoreactivity; positive cells/mm^2^)** | | | | |
| --- | --- | --- | --- | --- |
| **Treatment** | **Sex** | **Amygdala** | **Hippocampus** | **medial Caudal Nidopallium** |
| Non-predator | F | 321.5 | 253.5 | 121.4 |
| Non-predator | F | 370.6 | 238.1 | 81.2 |
| Non-predator | F | 350.4 | 226.6 | 134.5 |
| Non-predator | M | 499.8 | 290.7 | 153.1 |
| Non-predator | F | 369.2 | 331.0 | 151.0 |
| Non-predator | M | 368.8 | 298.1 | 109.7 |
| Predator | F | 534.7 | 406.5 | 149.8 |
| Predator | M | 510.2 | 394.6 | 154.9 |
| Predator | M | 726.2 | 293.3 | 140.5 |
| Predator | M | 803.2 | 478.5 | 123.0 |
| Predator | M | 589.9 | 419.6 | 152.1 |
| Predator | F | 481.7 | 381.9 | 137.8 |

**Supplementary Information**

**Predator-induced fear causes PTSD-like changes in the brains and behaviour of wild animals**

Liana Y. Zanette, Emma C. Hobbs, Lauren E. Witterick, Scott A. MacDougall-Shackleton & Michael Clinchy

| **Immediate neuronal activation data (cFos immunoreactivity; positive cells/mm^2^)** | | | |
| --- | --- | --- | --- |
| **Treatment** | **Sex** | **Amygdala** | **Hippocampus** |
| Non-predator | M | 586.3 | 179.4 |
| Non-predator | F | 938.3 | 284.6 |
| Non-predator | M | 761.1 | 196.7 |
| Non-predator | F | 575.0 | 214.2 |
| Non-predator | M | 350.0 | 169.5 |
| Non-predator | F | 306.8 | 229.6 |
| ‘Chick-a-dee’ | F | 732.5 | 463.7 |
| ‘Chick-a-dee’ | M | 335.9 | 355.0 |
| ‘Chick-a-dee’ | F | 606.7 | 370.0 |
| ‘Chick-a-dee’ | M | 670.5 | 318.5 |
| ‘Chick-a-dee’ | F | 600.1 | 192.8 |
| ‘High zee’ | M | 1504.3 | 502.9 |
| ‘High zee’ | M | 944.7 | 343.9 |
| ‘High zee’ | F | 596.1 | 362.3 |
| ‘High zee’ | F | 778.1 | 463.1 |
| ‘High zee’ | M | 802.4 | 529.0 |
| ‘High zee’ | F | 957.6 | 572.4 |
| Predator | M | 1588.4 | 486.3 |
| Predator | F | 1167.7 | 264.3 |
| Predator | M | 1031.1 | 255.2 |
| Predator | F | 972.5 | 371.0 |
| Predator | M | 933.0 | 292.3 |
